# Peritonitis: laparoscopic approach

**DOI:** 10.1186/1749-7922-1-9

**Published:** 2006-03-24

**Authors:** Ferdinando Agresta, Luigi Francesco Ciardo, Giorgio Mazzarolo, Ivan Michelet, Guido Orsi, Giuseppe Trentin, Natalino Bedin

**Affiliations:** 1 Department of General Surgery, Presidio Ospedaliero di Vittorio Veneto, Vittorio Veneto, (TV) Italy; 2 Via Borgo Coilsola, 1 31010 Fregona (TV), Italy

## Abstract

**Background:**

Laparoscopy has became as the preferred surgical approach to a number of different diseases because it allows a correct diagnosis and treatment at the same time. In abdominal emergencies, both components of treatment – exploration to identify the causative pathology and performance of an appropriate operation – can often be accomplished via laparoscopy. There is still a debate of peritonitis as a contraindication to this kind of approach. Aim of the present work is to illustrate retrospectively the results of a case-control experience of laparoscopic vs. open surgery for abdominal peritonitis emergencies carried out at our institution.

**Methods:**

From January 1992 and January 2002 a total of 935 patients (mean age 42.3 ± 17.2 years) underwent emergent and/or urgent surgery. Among them, 602 (64.3%) were operated on laparoscopically (of whom 112 -18.7% – with peritonitis), according to the presence of a surgical team trained in laparoscopy. Patients with a history of malignancy, more than two previous major abdominal surgeries or massive bowel distension were not treated Laparoscopically. Peritonitis was not considered contraindication to Laparoscopy.

**Results:**

The conversion rate was 23.2% in patients with peritonitis and was mainly due to the presence of dense intra-abdominal adhesions. Major complications ranged as high as 5.3% with a postoperative mortality of 1.7%. A definitive diagnosis was accomplished in 85.7% (96 pat.) of cases, and 90.6% (87) of these patients were treated successfully by Laparoscopy.

**Conclusion:**

Even if limited by its retrospective feature, the present experience let us to consider the Laparoscopic approach to abdominal peritonitis emergencies a safe and effective as conventional surgery, with a higher diagnostic yield and allows for lesser trauma and a more rapid postoperative recovery. Such features make Laparoscopy a challenging alternative to open surgery in the management algorithm for abdominal peritonitis emergencies.

## Background

Laparoscopy (LAPS) is becoming the preferred surgical approach to different pathologies due to the possibility of correctly diagnosing and treating them at one time [1-3,6,9,20,32]. There are undoubted advantages for patients thanks to this "gentle" and minimally invasive surgery, if compared to the open approach [7,14]. Especially if we take in consideration "emergency" abdominal situations, where both critical component of operative treatment (exploration to identify the causative pathology and performance of an appropriate operation) can often be "gently" accomplished Laparoscopically [1-3,6-8,17,18]. Peritonitis is still considered, by some authors, to be a contraindication to the Laparoscopic approach because the theoretical risk of enhanced bacteremia and endotoxemia by pneumoperitoneum [1,3,32]. We report herein the results of a retrospective analysis on a case-control series of Laparoscopic versus open emergencies procedures (OP) in peritonitis patients performed at our department from January 1992 to January 2002.

## Methods

From January 1992 to January 2002, a total of 935 patients (M: F = 407: 528; mean age 42.3 ± 17.2 years) underwent emergent and/or urgent abdominal surgical procedures. Among them, 602 (64.3%) were operated on Laparoscopically, of whom 112 (18.6%) with peritonitis. The diagnosis of peritonitis was based on the Laparoscopy and the finding of purulent fluid in the peritoneal cavity. Since minimally invasive surgery was not performed by all of the surgeons of our staff, patients admitted for acute abdomen were treated by Laparoscopy or open surgery according to the presence of a well-trained surgical team and not randomly allocated to either treatment. Furthermore, since the beginning of our experience we have decided not to use Laparoscopy in patients with history of previous abdominal malignancies, more than two major abdominal surgeries, massive bowel distension, and in those too ill to withstand pneumoperitoneum. The presence of diffuse peritonitis was not considered a formal contraindication to the use of a Laparoscopic approach. As a result, our series reflects a selection bias in favor of Laparoscopy as regards morbidity and mortality. Irrespective of the chosen approach, all patients underwent the same preoperative workup (chest X-rays, EKG, and routine blood tests). The outcome measures were the incidence of intraoperative complications, operative mortality (within 30 days from surgical procedure), postoperative morbidity and mortality rates. The statistical analysis was performed with the t-test for independent samples for continuous variables and the chi-square test or Fisher's exact test for categorical values. The level of significance was set at 5%.

About the technique: all patients underwent operation in the supine position under general anaestesia: bladder catheterization was carried out in relation to the age of the patients and the main pre-operative diagnosis. The first trocar has been always inserted with an open technique. Exploration of the peritoneal cavity was performed after introduction of the optic system through an umbilical port. Further ports were placed according to the nature of the disease. In the presence of diffuse peritonitis, the first step was to evacuate purulent collections and to perform abundant irrigation of the four abdominal quadrants with isotonic saline solution at 37 c using a high-flow irrigation-suction device. Once the diagnosis had been established, patients were managed Laparoscopically or underwent to a conversion to an open procedure. Drains were placed routinely at the end of the operation using the trocars position.

## Results

**1. Gastro-duodenal perforated ulcer: **of 51 patients admitted for a perforated gastro-duodenal ulcer, 25 (49%) (mean age 59,2 ± 14,5 years; range 28 ÷ 79) were approached via LAPS. The conversion rate was 12% and mainly due to inadequate ulcer localisation. The mean operating time was 90,2 ± 16,1 min. (range 60 ÷ 130 min) (OP: 63,4 ± 12,8 min; range 30 ÷ 100 min.) (p = ns) with a mean post-operative hospital stay of 11.3 ± 8.4 days (range 7 ÷ 28 days) (OP: 11.5 ± 6.7 days; range 7 ÷ 17) (p = ns). Morbidity was 16% (4 cases) (OP: 13.7%) (p = ns). We had, in the LAPS group, one post-operative death in a patient with history of ictus cerebri, with a post-operative fistula, who died of recurrent stroke.

**2. Suspected appendicitis and pelvic disease: **data refer to 370 out of 612 patients (mean age 23.2 ± 22.1 years; range 9 ÷ 65) who underwent LAPS for right lower quadrant abdominal pain. Of them 35 patients (9.4%) presented with a localized or generalised peritonitis. We had no mortality and no major intraoperative complications. Reinterventions were as high as 2.8% (1 case) in the LAPS group vs. nil in the OA group (p = ns). Conversion rate was 22.8% (8 cases) and due to dense adhesions. Postoperative complication rates were similar in LAPS and OP (2.8% vs. 0.8%; p = ns).

As regards the postoperative course, LAPS patients recovered more rapidly with a significantly shorter stay than OP patients (4.4 ± 1.2 vs 5 ± 3.40 days; p = 0.01) and flatus passing earlier (1.6 ± 0.7 vs 2.2 ± 1.2 days; p < 0.01;). Furthermore, LAPS patients experienced far less wound infections (nil vs 6.1%; p < 0.01).

**3. Small bowel obstruction: **out of 82 patients admitted to our institution for acute small bowel obstruction (SBO), 28 (34.1%) were approached by LAPS (mean age 56.4 ± 17.2 years, range 22 ÷ 79). They all presented with peritonitis (either localised or generalised). The average operating time was 45.1 ± 11.3 min (range 20 ÷ 65). Conversion to open surgery was required in 12 patients because of a trocar-borne visceral injury in one case; a required intestinal resection in 5 cases (due to severe ischaemia); impossibility to locate the disease in the remaining cases. On the whole, 57.1% (16 cases) patients were treated successfully with LAPS, with mortality and morbidity rates of 6.2% (OP: 12.1) (p = ns).

**4. Cholecystitis: **we have treated 165 patients admitted for acute cholecystitis (mean age 56.4 ± 12.7 years). Patients with peritonitis were 10 (6%). The conversion rate was 10% (1 cases) and it was due to dense adhesions and unclear anatomy. Morbidity and mortality were nil and the mean hospital stay 5.7 ± 2.3 days.

**5. Colonic perforations: **14 out of 25 patients (mean age 67.4 ± 18.3 years) underwent emergency Laparoscopic surgery for diffuse peritonitis secondary to perforated diverticular disease or iatrogenic perforation on colonoscopy (3 cases). The conversion rate was 14.2%. No oostomy was necessary. Neither major nor minor intraoperative adverse events were observed. The hospital stay lasted 7.2 ± 4.1 days on average (OP: 9.4 ± 5.6 days) (p = ns). There was no morbidity and mortality (OP morbidity = 22.2%) (p = ns). One patient underwent elective Laparoscopic sigmoid resection after 3.5 months.

## Discussion

Laparoscopy has gained widespread acceptance in common surgical practice as a diagnostic and therapeutic tool. Abdominal emergencies often pose a diagnostic challenge to the general surgeon [35,36]. A correct diagnosis is crucial because of the various diseases that may be responsible for the same symptoms, in order to plan the appropriate procedure or to avoid unnecessary Laparotomies. Non-invasive diagnostic procedures are expensive, not always conclusive and available in all settings [1,21,28,32]. Laparoscopy is the only minimally invasive technique to allow at the same time for adequate diagnosis, appropriate treatment and/or the best abdominal approach.

In 1992 we decided to approach abdominal emergencies with LAPS, if a well trained LAPS surgeon were present. Ever since that time, 602 patients admitted with acute abdomen have been approached Laparoscopically. Of them 112 presented with a frank acute abdomen due to peritonitis. The overall conversion rate was 23.2%; morbidity was 12.5% and mortality 1.76%. A definitive diagnosis was reached in 85.7% of patients, and 90.6% of these latter received proper treatment. Herein we wish to analyze the advantages of Laparoscopy in the management algorithm for acute abdomen due to peritonitis as regards its indications, morbidity, mortality and its socio-economic impact.

**1. Indications: **the absolute and relative contraindications to Laparoscopy in the treatment of abdominal emergencies are the same as for elective procedures [1-3,5,14,32]. As for peritonitis, there is a theoretical concern that the CO_2 _pneumoperitoneum may enhance bacteremia and endotoxemia due to the increased intraperitoneal pressure [25,32]. Despite contradictory reports, where in clinical trial of Laparoscopy versus conventional surgery different task (serum and local cytokine levels, cell mediated immunity, stress response hormones, bacteriemia and endotoxiemia; type of gas used – helium vs CO2, warming and humified surrounding, abdominal wall lifting, the intra-abdominal pressure used and so on) in different and mostly non comparable animal model are used, most clinical and experimental studies support the idea that Laparoscopy appears to produce a less inflammatory response with a less trauma and less tissue damage than the open one [25]. Acute phase reaction laboratory indicators, as ceruloplasmin, fibrinogen, hepatoglobin and alfa1 antityrpsin seems to be lower after Laparoscopy that op, as is for the neuroendocrine stress response and its metabolic consequence. Concerning the polymorphonuclear leukocites the OP presents major effect than the LAPS. Over the past few years there has been an increasing number of series on the use of Laparoscopy in the treatment of peritonitis reporting favorable results [4,10-13,15,16,19,22-24,27,29,31,33,34]. The only data about Laparoscopy still under suspicious are the length of the surgical procedure and a high intrabdominal pressure, which seem to have both a negative effect [1]. We do agree with the clinical practice guideline done by the EAES: *"...changes in systemic inflammatory and anti-inflammatory parameters...are less pronounced after Laparoscopic surgery than conventional surgery. Whether this leads to clinically relevant effects remain to be proven. There is no compelling clinical evidence that specific modifications of the pneumoperitoneum alter immunologic response...presupposing appropriate perioperative measures and hemodynamic stability, there are no contraindication to create a pneumoperitoneum when LAPSaroscopic surgery is applicable in cases of peritonitis*..."[25]. Last but not least it is to participate in the idea that in order to minimize post-surgical infection, beside the use of antibiotic prophylaxis, the main step is to optimize the immune response by maintaining homeostasis through nutritional support (especially the enteral way – and the LAPS assuring a faster GI recovery allows for an earlier enteral feeding!) and to the reduce the surgical trauma (as it is done by LAPS) which consequently reduces the stress response and immune suppression [1,25,32].

Surgical timing is another relevant issue: the earlier the better. As evidenced in acute cholecystitis, the degree of inflammation is strictly related to time from the onset of symptoms [1,14,35]. Generally speaking, We operated on 85% of our patients within 48 hours of admission with a consequent success rate of 95%. A similar success rate was observed in the group of perforated gastro-duodenal ulcers, where time to surgery was no longer than 24 hours.

**2. Diagnostic accuracy: **the diagnostic accuracy of LAPS was very high in our series (85.7), according to the rate (89%–100%) reported in the international literature [32]. The high diagnostic yield of LAPS is important especially in patients with pelvic disease – suspected appendicitis, were LAPS allows for a better thorough exploration of the abdominal cavity and identification of concomitant diseases than OP. In cases of unclear preoperative diagnosis, Laparoscopy can shorten the observation period and avoid the need for expensive laboratory and imaging test. [10]. About these last ones, the accuracy of radiography in these diseases reaches 75%, whereas the accuracy of abdominal ultrasound is 60–89%. The CT scan is more accurate (84–98%), but it is not always available in every hospitals situation. [30,32].

**3. Treatment options: **LAPS allows to perform the same surgical procedures as open surgery, or even to schedule the appropriate medical therapy in the presence of concomitant diseases. Another main advantages of Laparoscopic management of generalized peritonitis are a better quality of peritoneal washing and an easy cleaning in the deep abdominal areas (such as Douglas recessus), as well as minimal destruction of the abdominal wall. Another point: many patients with acute suppurative peritonitis do not have an obvious perforation, but rather an inflammatory and necrotic zone with edema and abscess formation. Therefore they can be safely treated with drainage near the pathology zone with a large peritoneal lavage and antibiotic therapy. This procedure may allows a second-stage Laparoscopic treatment of the underlying disease, such as a resection of a sigmoid diverticula in elective conditions. [12,13,29]. About the length of surgery is comparable to open surgery (we might consider the improvements in both equipment and the surgeon's learning curve). The time spent for treatment of diseases incidentally found at Laparoscopy should be weighted against the economic impact of a missed diagnosis [1-3].

**4. Conversion: **the most frequent causes of conversion were the presence of dense adhesions and unclear anatomy. Iatrogenic lesions are range second in frequency. A surgeon should never regard conversion as a defeat: using this approach it is possible to choose the most appropriate incision to treat the patient [5,6,8,10].

**5. Morbidity and mortality: **the results of our experience show the feasibility of LAPS in the treatment of abdominal emergencies with acceptable major morbidity and mortality rates, comparable to the ones reported for the OP [32]. The complications we observed occurred mainly at the beginning of our experience: undoubtedly they might be reduced by careful patient selection, increased skill, confidence, experience and familiarity of the surgical équipe with this technique. Concerning the minor morbidity (wound infection) the results are unquestionably in favour of LAPS when compared with OP. [11,14,32].

**6. Hospital stay: **hospital stay after LAPS is shorter when compared with open controls, and patients experience a faster recovery. [1,15-18,25].

**7. Costs: **the advantage of LAPS does not only consist of cosmesis but also of a decrease in operative trauma [22,24,31]. This latter results in a reduced incidence of wound infections and incisional hernias. Moreover the reduction in trauma aids in the patient's recovery. Thus it seems logical that – although the exact economic benefits of LAPS are difficult to assess – the earlier patients' recovery and return to work does benefit the whole society [9].

**8. Patient perception: **LAPS carries an unquestionably positive patient perception of surgery, thanks to its advantages (reduced postoperative pain, prompt recovery of gastrointestinal functions, shorter hospitalization, and improved cosmesis). As a consequence, there is an ever-growing request from the lay public.

**9. The surgeon: **last but not least. A small high pressure operating theatre and the wide variety of operative finding need a well-trained and experienced surgeon together with a well-trained team. In order to offer patients the same chance of cure, at our institution LAPS was performed only when a well trained laparoscopic surgeon was on call.

## Conclusion

On the basis of our experience LAPS in the treatment of abdominal emergencies due to peritonitis is possible, simple and reproducible, effective without any specific complications in experienced hands. LAPS provides a superior diagnostic accuracy as well as wider therapeutic potentials with an improvement of postoperative comfort than OP. Sparing patients unnecessary Laparotomies reduces postoperative pain, increases prompt recovery of gastrointestinal functions, shortens hospitalization, helps contain health-care costs and increases cosmesis. This approach appears to play a crucial role in the diagnostic and therapeutic algorithm in almost every abdominal emergency. On these grounds we advocate a wider adoption of Laparoscopy and are confident it will become more relevant in common surgical practice.
